# Supplementary material for: Complexation of Gold(III) with Pyridoxal 5′-Phosphate-Derived Hydrazones in Aqueous Solution
Source: Molecules. 2022 Oct 28;27(21):7346. doi: 10.3390/molecules27217346 (PMC9654535; doi:10.3390/molecules27217346)
Supplement: Supplementary file 1 [file molecules-27-07346-s001.zip › molecules-1995726-supplementary.pdf]

molecules

MDPI

# Supporting Information for

# Complexation of Gold(III) with Pyridoxal 5′-Phosphate-Derived Hydrazones in Aqueous Solution

**Natalia N. Kuranova, Daniil N. Yarullin, Maksim N. Zavalishin, George A. Gamov ***

Department of General Chemical Technology, Ivanovo State University of Chemistry and Technology, 153000 Ivanovo, Russia
* Correspondence: ggamov@isuct.ru; Tel.: +7-(915)-821-85-62

**Table S1.** Preliminary virtual screening results using PASS Online software [1].

| Compound | Strain | Disease | Confidence |
|---|---|---|---|
| **PLP-INH** | *RESISTANT Mycobacterium tuberculosis H37Rv* | Tuberculosis | 0.1909 |
| | *Enterococcus faecalis ATCC 29212* | Bacterial infection | 0.1762 |
| Complex $Au^{3+}$-**PLP-INH** | *RESISTANT Mycobacterium tuberculosis H37Rv* | Tuberculosis | 0.2552 |
| | *Enterococcus faecalis ATCC 29212* | Bacterial infection | 0.1930 |
| Complex $Au^{3+}$-**PLP-F2H** | *RESISTANT Mycobacterium tuberculosis H37Rv* | Tuberculosis | 0.1781 |
| **PLP-T2H** | *Dialister micraerophilus* | Periodontitis | 0.3580 |
| | *Dialister pneumosintes* | | 0.3580 |
| | *Dialister propionicifaciens* | | 0.2577 |
| | *Dialister invisus* | | 0.2190 |
| | *Mycobacterium mageritense* | Tuberculosis | 0.1647 |
| Complex $Au^{3+}$-**PLP-T2H** | *Dialister pneumosintes* | Periodontitis | 0.3812 |
| | *Dialister micraerophilus* | | 0.3812 |
| | *Dialister propionicifaciens* | | 0.2850 |
| | *Dialister invisus* | | 0.2349 |
| | *Mycobacterium mageritense* | Tuberculosis | 0.1582 |
| **PLP-T3H** | *Dialister invisus* | Periodontitis | 0.5628 |
| Complex $Au^{3+}$-**PLP-T3H** | *Dialister invisus* | Periodontitis | 0.5836 |

"AntiBac-Pred functional of PASS Online software allows user to predict the fact that chemical compound can inhibit the growth of one or more of 353 bacteria in concentration below the 10000 nM. The score for each compound is expressed as confidence in its activity, which is a difference between probabilities for chemical compound to inhibit and to do not inhibit the growth of the particular bacteria. The higher confidence means the higher chance of the positive prediction to be true.

Only activities with $P_a > P_i$ (confidence > 0) are considered as possible for a particular compound". The description is taken from [2].

Reference

1. Biomed. Chem.: Res. Methods, 2018, 1(1), e00004, doi: 10.18097/bmcrm00004
2. http://www.way2drug.com/antibac/

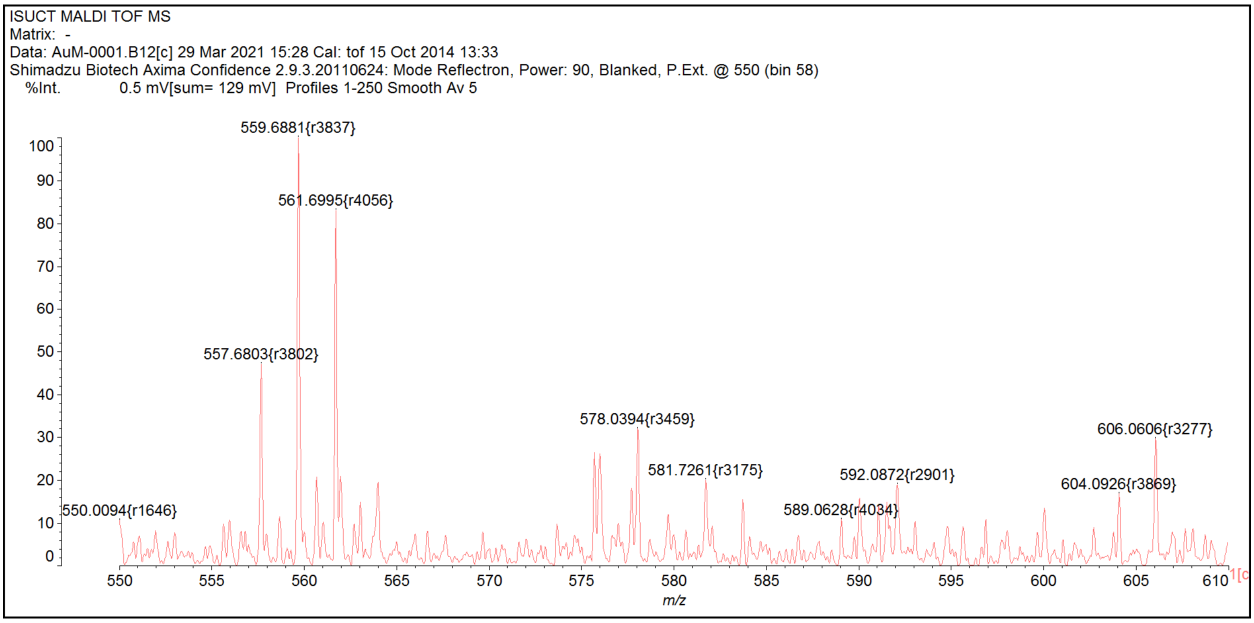


**Figure S1.** Mass-spectrum of Au-**PLP-INH** complex precipitated from an aqueous solution.

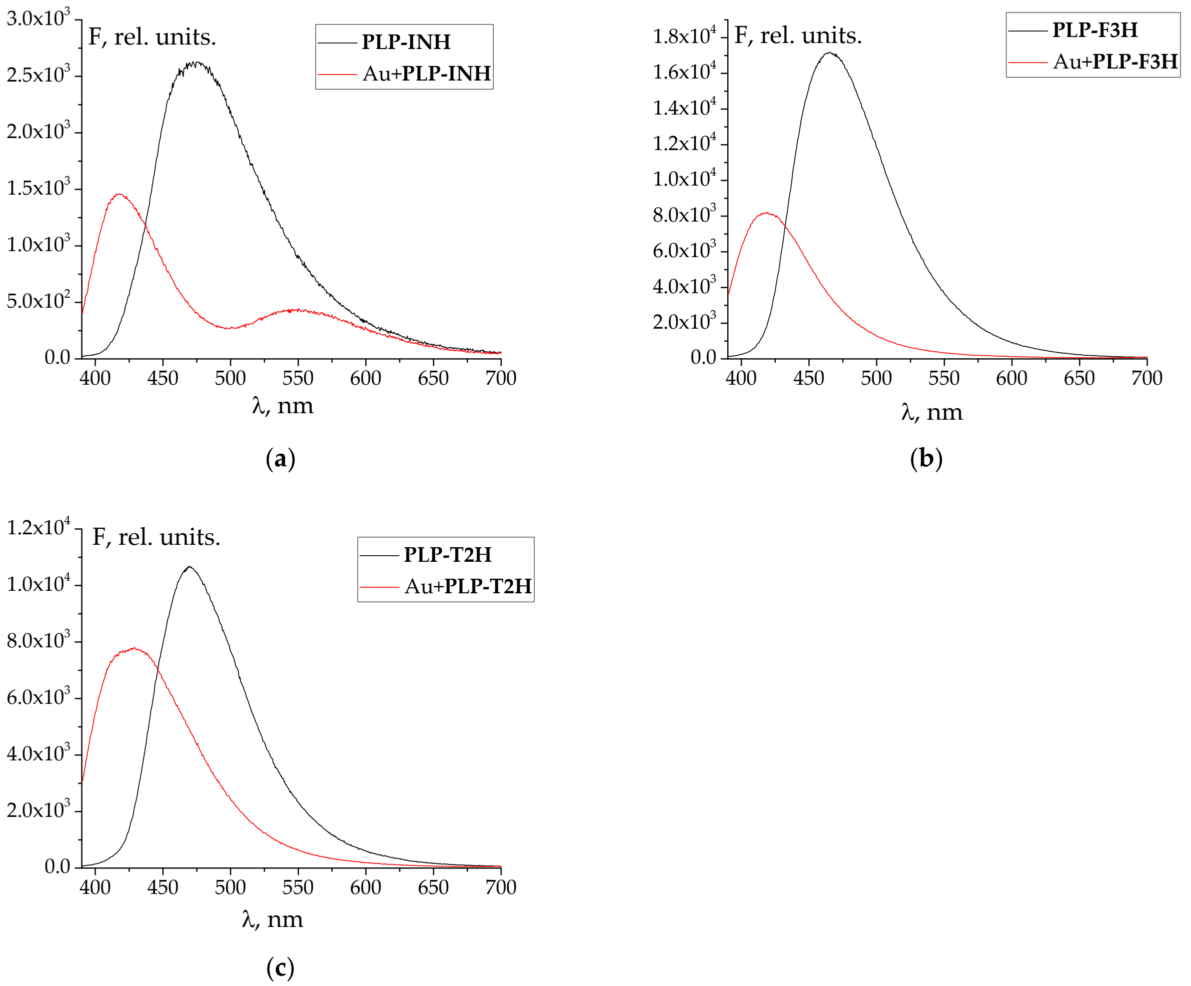


**Figure S2.** Emission spectra of free hydrazones and their mixtures with $Au^{3+}$: (**a**) **PLP-INH**; (**b**) **PLP-F3H**; (**c**) **PLP-T2H**. $\lambda_{ex}$ = 365 nm.

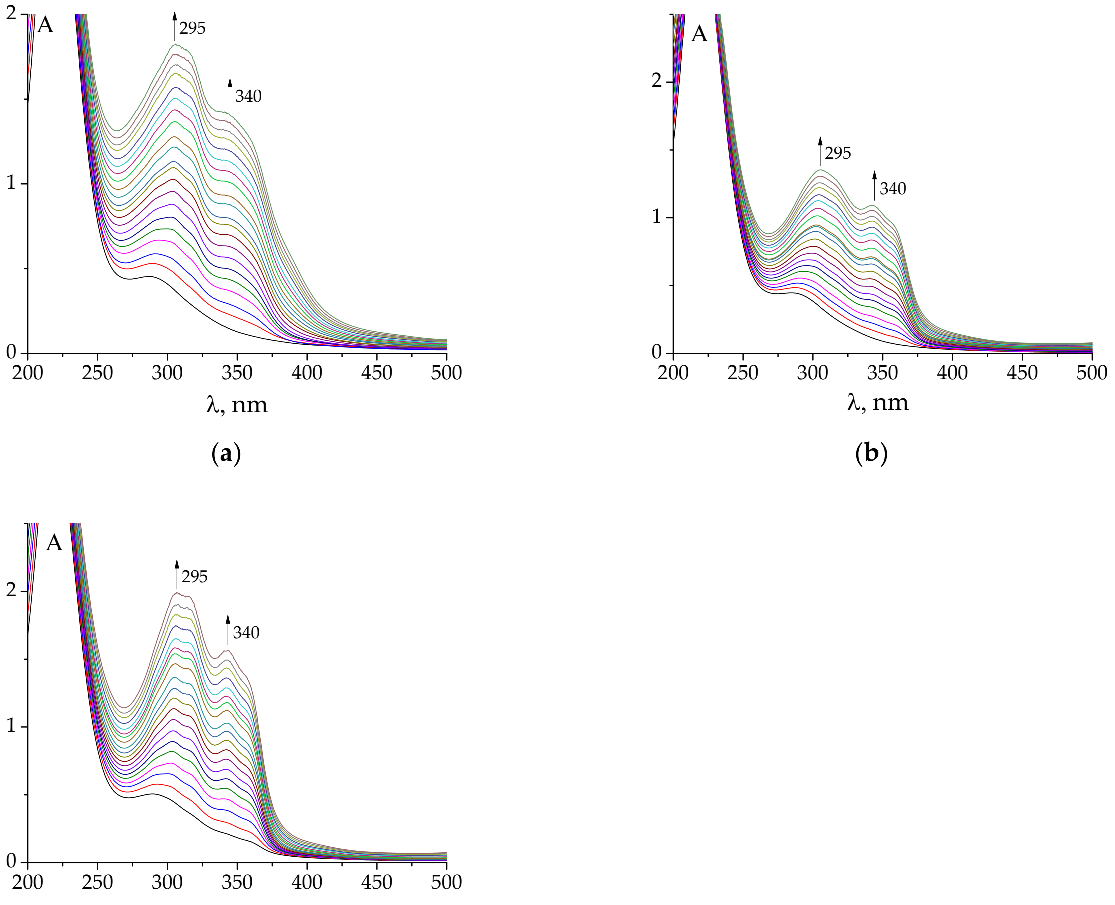


(**a**) (**b**) (**c**)

**Figure S3.** Examples of changes in UV-Vis spectra of $HAuCl_4$ upon addition of **PLP-INH** (**a**), **PLP-F3H** (**b**), **PLP-T3H** (**c**). Titrand: C($AuCl_4^-$) = $2{\cdot}10^{-4}$ mol $L^{-1}$; C($H^+$) = $5{\cdot}10^{-4}$ mol $L^{-1}$. Titrant: C(**PLP-INH**) = $1.5{\cdot}10^{-3}$ mol $L^{-1}$; C($OH^-$) = 0.01074 mol $L^{-1}$ (**a**); Titrant: C(**PLP-F3H**) = $1.5{\cdot}10^{-3}$ mol $L^{-1}$; C($OH^-$) = 0.01074 mol $L^{-1}$ (**b**); Titrant: C(**PLP-T3H**) = $1.5{\cdot}10^{-3}$ mol $L^{-1}$; C($OH^-$) = 0.01074 mol $L^{-1}$ (**b**). Twenty additions of 10 μL volume.

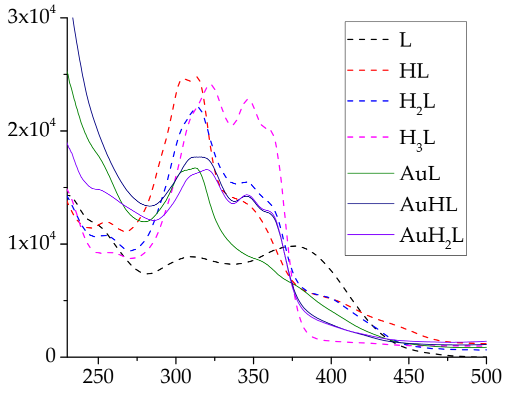


(**a**)

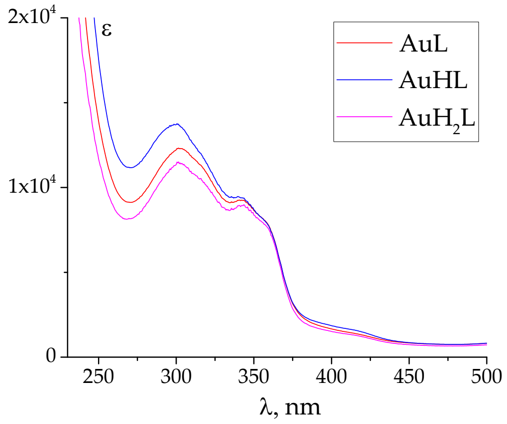


(**b**)

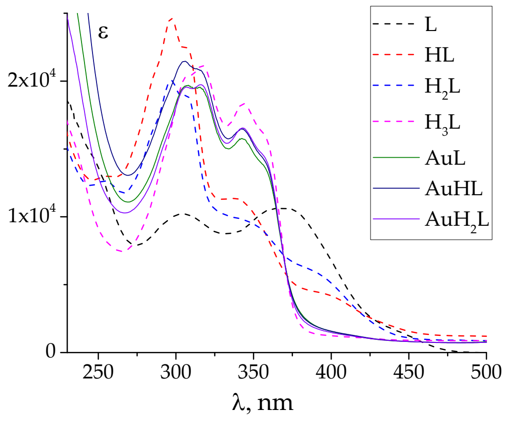


(**c**)

**Figure S4.** Calculated UV-Vis spectra of individual protonated and complex species of **PLP-T2H** (**a**), **PLP-F3H** (**b**), **PLP-T3H** (**c**). Spectra of protonated species are adopted from paper 10.1016/j.molliq.2020.112822.
